# Rubella, herpes simplex virus type 2 and preeclampsia

**DOI:** 10.1186/s12985-017-0813-6

**Published:** 2017-07-26

**Authors:** Shimos A. Alshareef, Ahmed M. Eltom, Abubakr M. Nasr, Hamdan Z. Hamdan, Ishag Adam

**Affiliations:** 1 Faculty of Medicine, University of Khartoum, P. O. Box 102, Khartoum, Sudan; 2 Department of Biomedical Sciences, King Faisal University, Alhasa, Saudi Arabia; 3 Al-Neelain Institute for Medical Research, Al-Neelain University, Khartoum, Sudan

**Keywords:** Rubella, Herpes simplex preeclampsia, Infections

## Abstract

**Background:**

Preeclampsia is a major health problem. Although, the pathophysiology of preeclampsia is not fully understood, there are recent studies on association between infections and preeclampsia.

**Objective:**

The aim of the present study was to investigate the association between maternal seropositivity of rubella, Herpes simplex virus type 2 (HSV-2) and preeclampsia.

**Method:**

A case –controls study (90 women in each arm) was conducted at Saad Abualila Maternity Hospital, Khartoum, Sudan.

The cases were women with preeclampsia and the controls were healthy pregnant women. Rubella and HSV-2 IgG antibodies were analysed in the maternal sera of all of the participants using ELISA.

**Results:**

There was no significant difference in the age, parity and gestational age between the two groups. Maternal serum IgG seropositivity for rubella (92.2% vs. 34.4%, *P* < 0.001) and HSV-2 (87.8% vs. 57.8%, *P* < 0.001) were significantly higher in preeclampsia than in the controls. There was no significant difference in the maternal serum IgM seropositivity for rubella (3.3% vs. 2.2%, *P* = 0.650) and HSV-2 (2.2% vs. 1.1%, *P* = 0.560). All the IgM seropositive cases were IgG seropositive too. In binary logistic regression women with rubella (OR = 4.93; 95% CI = 2.082–11.692, *P* < 0.001) and HSV-2 (OR = 5.54; 95% CI = 2.48–12.38, *P* < 0.001) IgG seropositivity were at higher risk for preeclampsia.

**Conclusion:**

In the current study rubella and HSV-2 IgG seropositivity is associated with preeclampsia. Preventive measure should be implemented.

## Background

Preeclampsia is defined by hypertension and proteinuria that occur in the second half of pregnancy. It may be a symptomless, however various signs and symptoms e.g. edema, visual disturbances, headache, and epigastric pain may be observed especially in the severe form of the disease [1]. Preeclampsia is major health problem where there are around 8.5 million cases of preeclampsia are reported worldwide annually and it complicates around 2–4% of deliveries [2, 3]. Preeclampsia/eclampsia is a main cause of maternal and perinatal morbidity and mortality [2, 4].

The exact pathophysiology of preeclampsia is not fully understood. However, there is a body of evidence of association between maternal infections and preeclampsia e.g. Rustveld et al.*,* reported that women with bacterial or viral infections were at two- fold higher risk of developing preeclampsia [5]. Other studies failed to document association between preeclampsia and infections such as *Chlamydia pneumonia*, *Herpes simplex virus 2* (HSV-2) [6].

Investigating the association between infection and preeclampsia is of paramount for clinicians and health planners because preventive measures and treatment could be employed to prevent preeclampsia and its undesirable adverse effects.

Preeclampsia/eclampsia is the main cause of maternal and perinatal mortality in Sudan [7, 8]. Moreover we have previously shown that 65.3% out of 231 pregnant women had anti-rubella-IgG positive [9]. The current study was conducted to investigate the association between rubella, HSV-2 and preeclampsia.

## Methods

A case –control study was conducted at Saad Abualila Maternity Hospital, Khartoum, Sudan during the period of February through July 2015. Saad Abualila Maternity Hospital is a tertiary teaching hospital governed by the Faculty of Medicine, University of Khartoum, Sudan.

The cases were women with preeclampsia (blood pressure ≥ 140/90 mmHg on 2 occasions, at least 6 h apart, and proteinuria of ≥300 mg/24 h). Cases were divided in mild and severe preeclampsia (blood pressure ≥ 160/110 mmHg on 2 occasions, at least 6 h apart, and proteinuria of ≥5 g/24 h); HELLP syndrome (hypertension, proteinuria and presence of hemolytic anemia, elevated liver enzymes and low platelet count [1]. The controls were pregnant women without hypertensive disorders, nephropathy, or diabetes or any underlying disease. Both the cases and the controls had singleton pregnancy.

After signing an informed consent, medical and obstetrics history (age, parity, and gestational age) were gathered using a questionnaire. The body mass index (BMI) was computed from the women’s weight in kg/height in square.

Five mL of blood was withdrawn, allowed to clot, centrifuged and kept at −20 °C until analysed. Specific rubella and HSV-2 antibody profiles were analysed using commercial rubella and HSV-2 -specific ELISA (Euroimmun, Lu¨beck, Germany) to detect seropositivity for IgM and IgG. The tests were performed as instructed by the manufacturer. The reagents have positive and negative controls in a ready to use solution that specific for HSV-2.

Results of ≥1.1 were considered positive, those in the range 0.9–1.1 were considered weakly positive and those ≤0.9 were interpreted as negative for the tested organisms (rubella and HSV-2).

The sample size was calculated as unmatched case control study where the expected prevalence of rubella IgG seropositivity was 83.0% vs. 65.0% in the cases vs. controls, respectively. This proportion was calculated depending on our previous finding of the prevalence (65.0%) of rubella IgG seropositivity among pregnant Sudanese women [9]. A sample of 90 women in each arm of the study have over 80% power to detect a difference of 5% at α = 0.05. We assumed that 10% of the women might have incomplete data or samples [10].

### Statistical analyses

The collected data were analysed using SPSS V.20.0 (SPSS Inc., Chicago, IL, USA). Student’s t test and χ^2^ tests were used to compare continuous and categorized data respectively. Binary regression analyses were performed where preeclampsia was the dependent variable and expected risk factors (age, parity, gestational age, education, etc.) and rubella and HSV-2 IgG seropositivity were the independent variables. Odds ratio (OR) with a 95% confidence interval (CI) was calculated and statistical significance was defined as *P* < 0.05.

## Results

Ninety women were enrolled in each arm of the study. There were 67 and 23 women with mild and severe preeclampsia, respectively. All the cases were late on set preeclampsia (> 34 weeks of gestation). While there was no significant difference in the age, parity, gestational age between the two groups, BMI was significantly higher and hemoglobin was significantly lower in the cases (preeclamptic women), Table 1. Maternal serum IgG seropositivity for rubella (92.2% vs. 34.4%, *P* < 0.001) and HSV-2 (87.8% vs. 57.8%, *P* < 0.001) were significantly higher in preeclampsia than in the controls. There was no significant difference in the maternal serum IgM seropositivity for rubella (3.3% vs. 2.2%, *P* = 0.650) and HSV-2 (2.2% vs. 1.1%, *P* = 0.560). All the IgM seropositive cases were IgG seropositive too.

**Table 1:** Basic characteristics of the case and controls

Variable	Cases (*n* = 90)	Controls (*n* = 90)	*P*
*Mean(SD)*			
Age, years	28.0 (5.6)	28.1 (4.7)	0.909
Parity	2.7 (1.5)	2.5 (1.6)	0.349
Gestational age, weeks	37.1 (1.9)	37.4 (1.3)	0.190
Body mass index, Kg/m^2^	25.6 (4.4)	24.3 (2.3)	0.012
Hemoglobin, g/dl	10.3 (1.4)	11.0 (1.3)	0.001
*Number (%)*			
Educational level < secondary	76 (84.4)	72 (80.0)	0.293
Housewives	84 (93.3)	82 (91.1)	0.799

In binary logistic regression women with rubella (OR = 4.93; 95% CI = 2.082–11.692, *P* < 0.001) and HSV-2 (OR = 5.54; 95CI = 2.48–12.38, *P* < 0.001) IgG seropositivity were at higher risk for preeclampsia, Table 2.

**Table 2:** Binary regression analyses of the predictors for preeclampsia

Variable	OR	95% CI	*P*
Age, years	1.01	0.92–1.10	0.800
Parity	1.23	0.92–1.65	0.149
Gestational age, weeks	0.88	0.69–1.13	0.337
Body mass index, kg/m^2^	1.10	0.94–1.28	0.222
Education	0.73	0.45–1.20	0.224
Housewives	0.80	0.23–2.70	0.721
Hemoglobin, g/dl	0.70	0.54–0.91	0.010
Rubella IgG seropositivity	4.93	2.082–11.692	< 0.001
HSV-2 IgG seropositivity	5.54	2.48–12.38	< 0.001

## Discussion

Our results have shown that women with rubella (OR = 4.93) and HSV-2 (OR = 5.54) IgG seropositivity were at higher risk for preeclampsia.

Recently Lao et al. have observed a significant association between rubella non-immunity with preeclampsia especially among multiparas women and preeclamptic women carrying a male fetus [11]. Likewise Schwartzenburg and colleuages have repeorted a significantly higher prevalence of preeclampsia (50%) in women with rubella non-immunity compared with low rate of preeclampsia among immune women (3.5%) [12].

In the current study women with HSV-2 IgG seropositivity were 5.5 times at higher risk for preeclampsia. In contrast previous reports have shown no association between HSV-2 and preeclampsia [5, 6, 13–15]. The difference in our study and the later ones could be explained by the genetic and immunological reactions of pregnant women to infections which might vary from one to another setting.

It is worth to be mentioned that the association between infections and preeclampsia might be setting-related.e.g. we have previously observed that women with infection (placental malaria) were 2.3 times at higher risk for preeclampsia [16].

As have been mentioned above, inflammatory response/process during pregnancy might have been initiated by infection, which can lead to endothelial dysfunction which is the main of feature of preeclampsia [17]. The leukocyte (mainly neutrophils) activation with increased production of reactive oxygen species and chemokines which are the features of normal pregnancy are exacerbated among preeclamptic women [18, 19].

Immune-modulation is another factor might compound the normal inflammatory response to pregnancy and increase the susceptibility to preeclampsia [20]. We have shown that IL-10 was significantly higher in the women with preeclampsia compared with pregnant healthy controls. Moreover, women with preeclampsia had significantly lower levels of IFN-gamma and IL-4 and significantly higher levels of IL-10 7 days later in comparison with the presenting levels [21]. This suggests that altered maternal cytokines may contribute to endothelial cell dysfunction and development of preeclampsia. Various infections (parasitic, bacterial or viral) can elicit the modulation of cytokines, leading to oxidative stress and endothelial dysfunction [17–19].

One of the limitations of the current study was the lack of follow-up from early pregnancy till the third trimester where a sero-conversion might have occurred. There were few recent infections (IgM) which beyond the statistical significance between the cases and controls. Hence the results interpretations were depend on IgG seropositivity. A larger longitudinal study is needed.

## Conclusion

In the current study rubella and HSV-2 IgG seropositivity is associated with preeclampsia. Preventive measure should be employed.

## Abbreviations

BMI: Body mass index
CI: Confidence interval
HSV-2: Herpes simplex −2
OR: Odds ratio
